# Aging at home: factors associated with independence in activities of daily living among older adults in Norway—a HUNT study

**DOI:** 10.3389/fpubh.2023.1215417

**Published:** 2023-10-04

**Authors:** Skender Redzovic, Beatrix Vereijken, Tore Bonsaksen

**Affiliations:** ^1^Department of Neuromedicine and Movement Science, Norwegian University of Science and Technology, Trondheim, Norway; ^2^Department of Health and Nursing Science, Inland Norway University of Applied Sciences, Elverum, Norway; ^3^Department of Health, VID Specialized University, Stavanger, Norway

**Keywords:** independence, activities of daily living, community-dwelling older adults, demographic factors, living situation factors, successful aging, Trøndelag Health Study

## Abstract

**Background:**

Maintaining independence in activities of daily living (ADL) is essential for the well-being of older adults. This study examined the relationship between demographic and living situation factors and ADL independence among community-dwelling older adults in Norway.

**Methods:**

Data was collected in Norway between 2017 and 2019 as part of the fourth wave of the ongoing Trøndelag Health Study (HUNT) survey, sent to all citizens in Trøndelag county over 20 years of age, which is considered representative of the Norwegian population. Included in the current cross-sectional study were 22,504 community-living individuals aged 70 years or older who completed the survey and responded to all items constituting the ADL outcome measure. Group differences in ADL independence were examined with Chi Square tests, while crude and adjusted associations with ADL independence were examined with logistic regression analyses. Statistical significance was set at *p* < 0.05.

**Results:**

The participants reported a high degree of independence in primary ADL and slightly lower in instrumental ADL. In the fully adjusted analyses, ADL independence was associated with lower age, female gender, higher levels of education and income, higher subjective well-being, having no chronic or disabling disease, and having someone to talk to in confidence. Surprisingly, women who were married had higher likelihood of ADL independence than unmarried women, whereas married men had lower likelihood of ADL independence than unmarried men.

**Conclusion:**

In addition to known demographic and disease-related factors, the social context affects independence in ADL even in a society that offers advanced health and homecare services to all older adults equally. Furthermore, the same social setting can have differential effects on men and women. Despite the healthcare system in Norway being well-developed, it does not completely address this issue. Further improvements are necessary to address potential challenges that older adults encounter regarding their social connections and feelings of inclusion. Individuals with limited education and income are especially susceptible to ADL dependency as they age, necessitating healthcare services to specifically cater to this disadvantaged demographic.

## Introduction

In Norway, as well as globally, the older adult population is increasing due to a rise in life expectancy (1). The current dependency ratio of the retired over the working part of the population is expected to have doubled by 2060 (2, 3), which will strain healthcare systems and increase public expenditures. To address this issue, Norwegian authorities have identified aging in place as a crucial strategy (4).

Aging in place refers to older adults living independently in their own homes, alone or with family (5). This idea has been broadened by the WHO to include remaining in the current community and living in the residence of one’s choice (6). Older adults typically prefer to age in place as it contributes to their well-being and promotes a sense of belonging and identity, as well as feelings of security (7, 8).

A relevant model for aging in place is the Competence-Press Model of aging that was introduced by Lawton and Nahemow (9), which envisions individuals possessing competences while the environment exerts demands or “press.” The model portrays the connection between personal competence and environmental press as an adaptation process yielding positive or negative outcomes. Competence is defined as the degree to which people are able to effectively interact with their situation, and encompasses five domains: biological health, functional health, cognition, time use, and social behavior (10). Lawton saw the first two as intrinsic to the individual’s physical state, while the latter three are more influenced by life experiences. Environmental press signifies contextual demands, emerging from five environmental domains: personal, group, supra-personal (cohort-related), social, and physical (natural or constructed environment). In more recent times, the Competence-Press Model of aging has been expanded to include the significant influence of social relationships and a sense of belonging among older adults (11, 12). Furthermore, empirical evidence has indicated a shift from seeking information to developing intimacy-centered social motivations as individuals grow older (13).

Gaugler et al. (14) reported that one of the main reasons for institutionalization of older adults is the loss of the ability to engage in activities of daily living (ADL). Activities of daily living are the essential basic routine activities that individuals perform daily to maintain their independence and ability to function, such as eating, bathing, going to the toilet (15), as well as instrumental activities of daily living (IADL), such as shopping, preparing food, housekeeping, and using transportation (16). ADL give structure to the day, provide a sense of meaning, and enhance independence and quality of life (17). Both physical and social environments have been shown to influence the independence of older adults in ADL (18–20).

Several studies have reported that aging is generally associated with a decrease in ADL independency (21–23). Higher levels of household income and education have been found to affect ADL independency positively (24). Gender has also been shown to play a role, with women having a higher probability of developing functional disabilities as they age than men, largely because they are prone to live longer. Older women have been reported to have lower functioning than older men in grooming, dressing, eating, ambulating, transferring, and going to the toilet (25). Another study found that older men between 60 and 80 years had better performance in ADL that require lower limb strength than age-matched women because they preserve leg strength longer (26). Furthermore, living situation in terms of whether older adults are married, separated, or divorced, have access to support when needed and someone to talk to in confidence, can influence ADL independency (22, 27).

Although older adults living in the community generally have sufficient levels of ADL abilities, demographic and living situation factors may play a crucial role in sustaining these abilities and preventing institutionalization (28, 29). This study seeks to investigate the relationship between demographic and living situation factors and independence in ADL among community-dwelling older adults in Norway.

Prior research has investigated several factors within individuals that may impact their performance in ADL, including factors such as physical, spiritual, cognitive, and emotional aspects (30). In addition, environmental factors such as the physical surroundings and social context have been explored (18–20). However, there are several areas where our knowledge is incomplete. Firstly, cultural and societal contexts surrounding older adults vary across geographical locations, and there is a shortage of large-scale investigations on this topic conducted in Europe, with none having taken place in Norway (19, 31, 32). Secondly, Norway takes pride in possessing one of the world’s most advanced government-funded healthcare systems. This system provides comprehensive care to all older adults where needed, which could decrease the need for practical assistance provided by social connections. Consequently, Norway is an interesting case to investigate how the social context influences ADL among older adults. Thirdly, aging in place may not necessarily be beneficial to older adults if appropriate support to facilitate community engagement is not provided (7, 33). Filling in these gaps in our knowledge may lead to new insights that may contribute to both practical applications and theoretical understandings in this field. This holds particular relevance for healthcare planning and the pursuit of promoting successful aging among older adults.

## Methods

### Study design

The data for this cross-sectional study was collected among older community-dwelling adults in Trøndelag county, Norway, between 2017 and 2019, as part of the fourth wave of the Trøndelag Health Study (HUNT4), which is one of the world’s largest and most comprehensive ongoing population-based health surveys (34). The population in Trøndelag county is considered representative of the Norwegian population (34), and all individuals living in the county who were over 20 years of age were invited to participate in the survey. The HUNT4 participation rate was high (*n* = 56.042, 54.0% in North Trøndelag and *n* = 107.711, 42.6% in South Trøndelag), indicating small risk of selection bias. However, participation was slightly higher among people aged 40–79 years, and lower among those who were unmarried and living in urban areas. The sample was ethnically homogeneous, limiting generalizability to people with non-European backgrounds (34).

### Participants

Of the 26,668 adults aged 70 years or older that completed the HUNT4 survey, 22,504 (84.4%) lived in their own home and responded to all items constituting the ADL outcome measure used in this study. These individuals were included in the data analyses. The sample consisted of 10,905 (48.5%) men and 11,599 (51.5%) women, with the larger proportion aged 70–74 years (*n* = 10,003, 44.4%). Approximately a third of the sample had higher education (*n* = 7,340, 32.9 valid %), and 12,619 (64.0 valid %) were married.

Participants filled out the questionnaires at the field station or their (nursery) home, after they had been informed about the study and provided consent. Study assessors were available in case of questions (34).

### Measures

#### Independence in activities of daily living

ADL independence was assessed on 16 discrete activities of daily living. Seven of the items were primary ADL (walk indoors, go to the toilet, wash the body, take bath or shower, get dressed/undressed, go to bed/get out of bed, eat) and the remaining nine items were instrumental ADL (cook a warm meal, do light housework, house cleaning, do the laundry, go shopping, pay the bills, take medications, go outside, take the bus). On each item, the participants indicated whether they were able or unable to perform the activity independently. A dichotomous variable was constructed based on the ADL items, where participants were classified according to their ability to perform all ADL independently (1) or not (0), and used as outcome variable in the analyses.

#### Sociodemographic variables

Age was collected as a continuous variable and categorized into 70–74 years, 75–79 years, 80–84 years, and 85+ years of age. Information was also collected about gender (male, female). For this study, educational level was categorized as elementary school, high school or vocational training, and higher education. Household annual income (in NOK) was categorized as less than 250,000, between 251,000 and 450,000, between 451,000 and 750,000, and above 750,000.

#### Health-related variables

Perceived health was assessed with one item: “How is your health at the moment?” Response options were “poor,” “not so good,” “good,” and “very good.” Higher scores indicated better perceived health. The participants were also asked whether they had a chronic and disabling disease or injury, with response options “yes” and “no.”

#### Living situation variables

The participants indicated whether they were married, unmarried, widow(er), separated, divorced, or did not want to respond. Subsequently, a dichotomous marital status variable was created by distinguishing between those reporting to be married and those who reported otherwise.

Social support was measured with the question: “Do you have someone who can provide help when you need it?” Response options were “yes” and “no.” Closeness was measured with the question: “Do you have someone you can talk to in confidence?” Response options were “yes” and “no.”

No secondary data was used in the current study.

### Statistical analysis

Individuals with missing scores on variables other than the ADL measures were removed casewise. Missing values constituted between 0 and 12.6% of the total amount of data, with only two variables containing more than 3% missing values (i.e., household income [3.8%] and marital status [12.6%]). ADL independence was defined as having an ADL score of 16, indicating the ability to perform all specified activities independently.

ADL independence was cross tabulated with all independent variables, and group differences in proportions with full ADL independence were examined with Pearson’s Chi Square coefficient. We checked for multicollinearity among the explanatory variables by examining bivariate correlations between conceptually related variables (marital status, social support, closeness). Social support and closeness were associated with a small to medium effect size (*r* = 0.25, *p* < 0.001), otherwise correlations were between 0.09 and 0.10.

Univariate and multivariate logistic regression analyses were performed to obtain effect sizes (odds ratio, OR) for the unadjusted and adjusted associations between each of the independent variables and ADL independence. Thus, each OR indicates the change (increase or decrease) in likelihood for being classified as ADL independent by each increase in the relevant independent variable. Possible interactions were examined post-hoc in logistic regression analyses. Statistical significance was set at *p* < 0.05.

## Results

### Independence in activities of daily living

The number and proportions of the sample being independent in each of the ADL are displayed in Table 1. Independence in primary ADL ranged between 97.2% (wash the body, take a bath/shower) and 99.2% (eat). For instrumental ADL, independence ranged between 88.7% (house cleaning) and 98.5% (take medications). Full ADL independence was reported by 18,101 (80.4%) of the participants, and the mean ADL score (range 0–16) was 15.5 (SD 1.6). ADL independence in different sample subgroups is shown in Table 1. Among those reporting being not fully independent in all ADL (*n* = 4,403, 19.6%), the mean ADL score was 13.2 (SD 2.7).

**Table 1:** Independence in each of the activities of daily living (ADL; *n* = 22,504).

ADL	Not independent	Independent
	*n* (%)	*n* (%)
Primary ADL		
Walk indoors	259 (1.2)	22,245 (98.8)
Go to the toilet	235 (1.0)	22,269 (99.0)
Wash the body	354 (1.6)	22,150 (98.4)
Take a bath/shower	621 (2.8)	21,883 (97.2)
Get dressed/undressed	321 (1.4)	22,183 (98.6)
Go to bed/get out of bed	272 (1.2)	22,232 (98.8)
Eat	178 (0.8)	22,326 (99.2)
Instrumental ADL		
Cook a warm meal	605 (2.7)	21,899 (97.3)
Do light housework	389 (1.7)	22,115 (98.3)
House cleaning	2,553 (11.3)	19,951 (88.7)
Do the laundry	1,446 (6.4)	21,058 (93.6)
Go shopping	901 (4.0)	21,603 (96.0)
Pay the bills	1,413 (6.3)	21,091 (93.7)
Take medications	339 (1.5)	22,165 (98.5)
Go outside	476 (2.1)	22,028 (97.9)
Take the bus	1865 (8.3)	20,639 (91.7)

### ADL independence in sample subgroups

Independence in all ADL was reported among larger proportions of individuals in the younger age groups, among women, and among those with higher levels of education and household income. Similarly, independence in all ADL was more frequently reported among individuals with better perceived health, and among those who reported no chronic or disabling disease or injury during the past year. Independence in all ADL was also more frequently reported among individuals who were married and individuals who had someone they could talk to in confidence (see Table 2).

**Table 2:** ADL independence by sample subgroups.

Characteristics	Not independent in all ADL	Independent in all ADL	*p*
Sociodemographic variables	*n* (%)	*n* (%)	
Age			
70–74 years	979 (9.8)	9,024 (90.2)	<0.001
75–79 years	1,272 (18.2)	5,732 (81.8)	
80–84 years	1,059 (30.2)	2,451 (69.8)	
85 + years	1,093 (55.0)	894 (45.0)	
Gender			
Male	2,222 (20.4)	8,683 (79.6)	<0.01
Female	2,181 (18.8)	9,418 (81.2)	
Education level			
Elementary school	2,421 (24.1)	7,623 (75.9)	<0.001
High school/vocational training	966 (19.5)	3,991 (80.5)	
Higher education	982 (13.4)	6,358 (86.6)	
Household income (NOK)			
< 250,000	977 (31.3)	2,145 (68.7)	<0.001
250,000-450,000	1808 (21.2)	6,719 (78.8)	
451,000-750,000	1,138 (15.3)	6,318 (84.7)	
>750,000	264 (10.0)	2,383 (90.0)	
Health-related variables			
Perceived health			
Poor	366 (78.9)	98 (21.1)	<0.001
Not so good	2,249 (34.7)	4,234 (65.3)	
Good	1,527 (11.9)	11,308 (88.1)	
Very good	119 (5.5)	2030 (94.5)	
Chronic and disabling disease			
Yes	3,544 (28.7)	8,794 (71.3)	<0.001
No	782 (8.0)	9,003 (92.0)	
Living situation			
Marital status			
Not married	1,553 (21.9)	5,550 (78.1)	<0.001
Married	2,364 (18.7)	10,255 (81.3)	
Social support			
Someone can provide support	4,203 (19.4)	17,498 (80.6)	0.05
No-one can provide support	129 (22.7)	440 (77.3)	
Closeness			
Someone to talk to in confidence	4,048 (19.2)	17,046 (80.8)	<0.001
No-one to talk to in confidence	278 (25.2)	823 (74.8)	

Missing values range between 0 and 12.4%. Not married implies either unmarried, separated, divorced, or widow(−er). Independent in all ADL implies a score of 16 on the ADL scale (range 0–16). Not independent in all ADL implies a score in the 0–15 range. *p* values are derived from Chi Square tests of independence.

### Multivariate associations with activities of daily living independence

Results from the univariate and multivariate logistic regression analyses are displayed in Table 3. The multivariate model was statistically significant (*p* < 0.001) with pseudo *R*^2^ ranging between 0.18 (Cox & Snell) and 0.29 (Nagelkerke). Individuals of higher age were significantly less likely to report ADL independence compared to their younger counterparts, whereas females were more likely ADL independent than men. Individuals with higher levels of education had higher likelihood of being ADL independent, compared to individuals with elementary education. Higher levels of household income were associated with ADL independence. Similarly, better perceived health and reporting not having a chronic or disabling disease or injury were associated with higher likelihood of ADL independence.

**Table 3:** Logistic regression analyses showing univariate and multivariate associations with ADL independence (multivariate model: *n* = 18,146).

Independent variables	Unadjusted associations			Adjusted associations		
	OR	95% CI	*p*	AOR	95% CI	*p*
Sociodemographic						
Age						
70–74 years	11.27	10.09–12.58	<0.001	8.75	7.61–10.06	<0.001
75–79 years	5.51	4.95–6.13	<0.001	5.14	4.49–5.87	<0.001
80 + years	2.83	2.53–3.17	<0.001	2.75	2.39–3.16	<0.001
85 + years (ref.)	–	–	–	–	–	–
Gender						
Female	1.11	1.04–1.18	<0.01	1.33	1.22–1.45	<0.001
Male (ref.)	–	–	–	–	–	–
Education level						
Higher education	2.06	1.90–2.23	<0.001	1.39	1.24–1.56	<0.001
High school/vocational	1.31	1.21–1.43	<0.001	1.14	1.02–1.27	<0.05
Elementary (ref.)	–	–	–	–	–	–
Household income (NOK)						
> 750,000	4.11	3.55–4.77	<0.001	1.63	1.32–2.01	<0.001
451,000–750,000	2.53	2.29–2.79	<0.001	1.39	1.20–1.60	<0.001
250,000–450,000	1.69	1.54–1.86	<0.001	1.30	1.15–1.47	<0.001
< 250,000 (ref.)	–	–	–	–	–	–
Health-related						
Subjective well-being (cont.)	3.95	3.72–4.19	<0.001	2.96	2.74–3.19	<0.001
Chronic and disabling disease						
No	4.64	4.27–5.04	<0.001	1.79	1.61–1.99	<0.001
Yes (ref.)	–	–	–	–	–	–
Living situation						
Marital status						
Married	1.21	1.13–1.30	<0.001	0.75	0.68–0.83	<0.001
Not married (ref.)	–	–	–	–	–	–
Social support						
Someone can provide support	1.22	1.00–1.49	0.05	0.91	0.70–1.18	0.46
No-one can provide support (ref.)	–	–	–	–	–	–
Closeness						
Someone to talk to in confidence	1.42	1.24–1.64	<0.001	1.34	1.12–1.61	0.001
No-one to talk to in confidence (ref.)	–	–	–	–	–	–
Model parameters						
Hosmer Lemeshow test, *p*					8.83	0.36
Model Chi Square, *p*					3,587	<0.001
Cox & Snell *R*^2^					0.18	
Nagelkerke *R*^2^					0.29	

The adjusted model shows associations adjusted for all variables in the model. In the adjusted model, 4,358 cases had one or more missing values on the included variables, and these cases were removed from the analysis.

Of the living situation variables, being married was associated with higher likelihood of ADL independence in the unadjusted analysis, while this association was reversed in the multivariate analysis. Reporting having social support was unrelated to ADL independence in both unadjusted and adjusted analyses. Having someone to talk to in confidence was related to higher likelihood of ADL independence.

### *Post-hoc* interaction analyses

The effect of gender on ADL independence was larger in the adjusted model compared to the unadjusted model, whereas the association between marital status and ADL independence was reversed when adjusting for all variables. To explore a possible explanation for these results, we examined the interaction between gender and marital status in explaining ADL independence.

A logistic regression model including gender and marital status as the only independent variables revealed a statistically significant association between female gender and ADL independence, and also between being married and ADL independence. When included in the second step, the interaction term gender × marital status was significant, suggesting that the association between marital status and ADL independence was dependent on gender. Hence, we examined the associations between marital status and ADL independence separately for men and women. Among men, being married was associated with lower odds of ADL independence (OR: 0.88, 95%CI: 0.78–0.99, *p* < 0.05). In contrast, among women, being married was associated with higher odds of ADL independence (OR: 1.66, 95%CI: 1.51–1.94, *p* < 0.001).

## Discussion

This study investigated the relationship between demographic and living situation factors and independence in ADL in community-dwelling older adults in Norway. Consistent with earlier research in this field, the results indicated that independence in ADL is associated with younger age, female gender, higher education, higher household income, better perceived health, and having someone to talk to in confidence. Surprisingly, the association between marital status and ADL independence was dependent on gender, with being married associated with lower likelihood of ADL independence among men, but higher likelihood of ADL independence among women.

In recent decades, reports have indicated that the prevalence of ADL and IADL disability among people in the older population ranges between 11 and 49% (23, 35), but that disability may be partly reversible with adequate support (23). Not surprisingly, the current study confirmed that community-dwelling older adults in Norway generally have a high degree of independence in ADL, but that independence decreases with age. ADL dependency was more commonly reported by participants in their 80s, which further accelerated at age 85+. This is also consistent with previous research (22, 23, 35), although it seems that the prevalence of ADL dependency is higher in Norway than in other comparable countries. For instance, only 48% of individuals in Sweden who were 85 years old in 2015 reported ADL dependency, compared to 55% in the current study. However, differences in study design, definitions, and methods used to measure ADL independence may partly explain some of the variation.

A larger proportion of study participants reported needing help with IADL, such as with cleaning, using public transportation, managing finances, doing laundry, and grocery shopping. This suggests higher environmental “press” in these activities that demand higher levels of physical and cognitive functioning, and are thus more challenging to maintain as people age (9). Many older adults reside in homes, communities and environments that may not be suitable for declining functional abilities. Recent studies reported that having more age-friendly features in a community was associated with a greater perception of age-friendliness by older adults and a stronger desire to age in place. Moreover, higher perceived accessibility to services and sites was associated with greater quality of life (20, 36).

The findings in the present study highlight that both health state, health perception, education and income are major factors associated with an independent life and aging in place, which is consistent with previous studies (24, 30, 37). However, for some groups in particular, individual proficiency may not align with external pressures and result in unfavorable consequences for their ADL independency (9). For example, older adults with lower education and income may have had more strenuous working tasks, limited purchasing power, fewer leisure activities, and poorer health literacy and access to health services, which can all contribute to a larger burden on their health and functioning over time.

In the current study, women had higher likelihood of ADL independency compared to men. Although existing evidence is mixed with regard to gender differences in ADL disability (38), most studies have reported no differences or women having higher likelihood of ADL dependency (25, 26, 39, 40). Given that women have been found to have higher rates of functional impairment as measured by both clinical tests and self-reports (37, 41), our findings are interesting and may point to positive effects of Norway’s longstanding efforts to promote gender equality by enhancing women’s rights as related to education, income and work opportunities (42). This development may have strengthened women’s personal resources and enhanced their ability to cope with environmental demands at older age (9).

One of the most interesting findings in the present study is that having someone to confide in was linked to greater ADL independence. This is in line with previous findings that social and emotional support are important for older adults to maintain their independence in ADL and supports the notion that meaningful contacts and relationships are important for ageing well (11, 19, 31, 32, 43–45). Nevertheless, a number of these impacts remain unclear. The variable “closeness” can be interpreted as a measure of whether they feel socially and emotionally connected to someone else, but can also encompass a practical dimension as closeness to others gives access to support. Thus, older adults’ social situation can positively or negatively influence their ADL independence (46, 47). On the other hand, assistance from health services (i.e., being dependent in ADL), which is provided to all citizens in Norway regardless of their income, may also help in alleviating burdensome feelings, as previous studies have reported a statistically significant relationship between receiving support in carrying out ADL and lower levels of social and emotional loneliness, in addition to having frequent social interactions with family, friends, or neighbors (46, 48). Environmental barriers, migration patterns, unsafe neighborhoods, inaccessible housing, inadequate resources for socializing, the role of recent losses of family and friends, as well as mental health issues are reported to contribute to loneliness in older adults (49). In contrast, social connection in older adults is a predictor of good physical and mental health and their quality of life (50).

Studies conducted in Western societies have shown that living arrangement (i.e., living with a partner or not) can affect morbidity to a larger extent than marital status. An earlier study found that individuals living with a partner had lower morbidity rates compared to those living alone, and that excess risk of illness among the never married, widowed, and divorced was reduced by 40–70% for most health measures after controlling for living arrangement (51). These findings indicate that health effects of marital status are largely tied to the inherent living arrangement, with people in paired living arrangements without being married experiencing similar health benefits as those who are married. Our study showed that being married was associated with higher likelihood of ADL independence among women, while the opposite was the case for men. In contrast, another study showed that women living alone had less chance of experiencing a decline in functional status than those living with spouses or others (52). Our findings underscore the significance of understanding the role of relationships in the aging journey. Many aspects of relationships are interconnected, and gender differences concerned with caring attitudes among older people may play a part (53). Possibly, older women may be inclined to identify with a caring role, indicating that their motivation to care for others may help them maintain ADL independence - not just for their own sake, but also for the benefit of their spouse. Conversely, older men who are married may be less inclined to maintain ADL independence if their needs can be taken care of by their wife. On the other hand, older men who are not cared for by a spouse in the event of functional decline may have a stronger drive toward maintaining ADL independence. Nonetheless, the challenge of social relationships is to optimize the advantages that offer protection while minimizing any unfavorable negative factors (11). Further research is needed to investigate the implications of marital status and living in a paired relationship for men and women in greater detail.

While the results of the current study confirm and further validate previously established scientific understanding, the present study provides several insights to existing knowledge. Firstly, despite the evidence that social surroundings impact ADL and IADL, there is a scarcity of large-scale studies conducted in Europe, and none has been conducted in Norway (19, 31, 32). The social environment surrounding older adults is deeply rooted in the cultural fabric of a society, potentially assuming diverse roles in different geographical locations. Secondly, Norway stands out as a country with one of the world’s most advanced government-funded healthcare systems. This comprehensive system caters to the needs of all older adults equally, which could reduce the need for social connections to provide practical assistance in daily life. Nevertheless, our findings indicate that even in a society like the one in Norway, social context is important for ADL independency in older adults. Moreover, our results indicate that similar social circumstances can yield different effects on men and women. Thirdly, our results indicate that further efforts are needed to identify and provide support to those susceptible to losing independence, which constitutes a vital aspect for healthcare planning and the advancement of successful aging for older adults.

### Implications for practice and policy

Older adults place high importance on maintaining ADL independence. Therefore, it is crucial to identify modifiable factors that contribute to the onset of ADL disability and can inform the development and employment of support mechanisms.

As longevity increases, home care services need to be designed to meet increasing needs for support from community-dwelling older individuals, especially those who are 85 years or older.

Special attention should be paid to supporting independence in ADL for community-dwelling individuals with lower education and income, as this can contribute to an active and longer independent life, lower demands for home care services, and lower expenditures. A recommendation from this study involves directing special attention toward aiding independence in ADL for those who lack confidants. Furthermore, these findings indicate that even in a fully established homecare system within an egalitarian welfare state such as Norway, there is a requirement for additional development to tackle the potential challenges faced by older adults concerning their relationships and sense of belonging. In turn, this enhancement may serve to maintain older people’s independence in ADL.

### Study strengths and limitations

The main strengths of this study are a large and representative sample of Norwegian older adults and access to a wide range of variables that may be relevant for ADL functioning.

The outcome measure (ADL independence) might be challenging to define as it encompasses large variation; some individuals may have difficulty with one activity while others struggle with multiple activities. At the same time, what one is capable of doing is rarely a fixed capacity across different situations, and factors such as daily form and context may affect independence but were not taken into account. Furthermore, while “marital status” most often implies living with a partner, “not married” can obscure whether people are in fact living in a paired relationship with another person. Thus, results related to marital status should be treated with some caution. Social support, closeness, and perceived health status were all measured using single-item scales. Thus, the constructs underpinning these measures may be somewhat loosely defined and subject to the respondents’ interpretation.

Generally, missing data is a threat to the validity of results, and sophisticated methods for overcoming problems with missing data have been developed in recent years (54). However, while the case-wise deletion method used in the present study may increase the risk of biased results by excluding participants with missing data, we considered that the very large sample size and the relatively small sample proportions with missing data (less than 3% on all except for two variables) constituted only a modest risk of skewed results.

As our study is cross-sectional, we are unable to identify factors that may impact ADL independency over time. In addition, we cannot establish the direction of statistical relationships. Nevertheless, this study identified focus areas that can be examined longitudinally across multiple HUNT surveys in future research.

## Conclusion

This study has highlighted several demographic and living situation factors that are significantly associated with independence in ADL among community-dwelling older people in Norway, namely age, gender, education, household income, perceived health, marital status and having someone to talk to in confidence. People with lower levels of education and income are at particular risk of experiencing ADL dependency in older age, and policymakers and healthcare services should target this disadvantaged group. Additionally, the study’s most intriguing results indicate that the social context holds influential and enduring impacts on independence in ADL even in a society where advanced health- and homecare services are provided to all older adults in need. Furthermore, similar social environments can exert varying influences on men and women. Even though health services in Norway are highly developed, they might not fully tackle this concern. Taking proactive measures, such as selecting, compensating, and optimizing interactions with relationship partners, can have a favorable influence on the aging process and on effectively managing the challenges associated with growing older.

## Data availability statement

The data analyzed in this study was obtained from the Trøndelag Health Study (HUNT; http://www.ntnu.edu/hunt/data). The following licenses/restrictions apply: the data are stored in the HUNT Databank and biological material are stored in the HUNT Biobank. The HUNT Research Centre has permission from the Norwegian Data Inspectorate to store and handle these data. The key identification in the data base is the personal identification number given to all Norwegians at birth or immigration, while de-identified data are sent to researchers upon approval of a research protocol by the Regional Ethical Committee and the HUNT Research Centre. To protect participants’ privacy, the HUNT Research Centre aims to limit storage of data outside of the HUNT Databank, and cannot deposit data in open repositories. The HUNT Databank has precise information on all data exported to different projects and are able to reproduce these on request. There are no restrictions regarding data export given approval of applications to the HUNT Research Centre. Requests to access these datasets should be directed to the HUNT Research Centre, kontakt@hunt.ntnu.no.

## Ethics statement

The Regional Committee for Medical Research Ethics in Norway approved the study (Project ID 186668).

## Author contributions

SR, BV, and TB contributed substantially to the conceptualization and design of the study. TB performed the statistical analysis and reported the findings. All authors contributed to the interpretation of the data. SR wrote the first draft. BV and TB revised it critically for important intellectual content. All authors contributed to the article and approved the submitted version.
